# Zika Virus Inhibitors Based on a 1,3-Disubstituted 1*H*-Pyrazolo[3,4-*d*]pyrimidine-amine Scaffold

**DOI:** 10.3390/molecules27186109

**Published:** 2022-09-19

**Authors:** Eunkyung Jung, Ruben Soto-Acosta, Robert J. Geraghty, Liqiang Chen

**Affiliations:** Center for Drug Design, College of Pharmacy, University of Minnesota, Minneapolis, MN 55455, USA

**Keywords:** Zika virus, flavivirus, antiviral agents, Zika virus inhibitors

## Abstract

To search for Zika virus (ZIKV) antivirals, we have further explored previously reported 7*H*-pyrrolo[2,3-*d*]pyrimidines by examining an alternative substitution pattern of their central scaffold, leading to compound **5** with low micromolar antiviral activity. To circumvent the synthetic difficulties associated with compound **5**, we have exploited a 1*H*-pyrazolo[3,4-*d*]pyrimidine scaffold and performed structure-activity relationship studies on its peripheral rings A and B. While ring B is less sensitive to structural modifications, an electron-withdrawing group at the para position of ring A is preferred for enhanced antiviral activity. Overall, we have not only discovered an alternative substitution pattern centered on a 1*H*-pyrazolo[3,4-*d*]pyrimidine scaffold but also generated anti-ZIKV compounds including **6** and **13**, which possess low micromolar antiviral activity and relatively low cytotoxicity. These compounds represent new chemotypes that will be further optimized in our continued efforts to discover anti-ZIKV agents.

## 1. Introduction

Zika virus (ZIKV) was first isolated in and named after the Zika forest of Uganda in 1947 [1]. The first human cases of ZIKV infection were reported in 1952 in the United Republic of Tanzania and Uganda, followed by outbreaks on the Yap Islands in Micronesia in 2007 and French Polynesia in 2013 [2]. However, it was the 2015–2016 global epidemic that attracted public attention and led to the declaration of ZIKV as a global public health emergency by the World Health Organization (WHO) in 2016 [3]. This declaration was in part due to the finding that ZIKV infection was associated with devastating neurological disorders, including congenital microcephaly in newborn infants and Guillain-Barré syndrome in adults [4]. As of December 2021, 89 countries have reported local transmission but currently no outbreaks have been reported [5]. Because the virus is still circulating in these areas and there is an increase in the mosquito-circulating areas due to overpopulation and urbanization, the risk for a new Zika virus is still relevant. 

ZIKV is a member of the genus *Flavivirus*, which also includes important mosquito-borne viruses such as dengue virus (DENV), West Nile virus (WNV), yellow fever virus (YFV), and Japanese encephalitis virus (JEV) [6]. Like other *Flavivirus* members, ZIKV virions contain a single-stranded, positive-sense genomic RNA, which is translated into a single polyprotein and subsequently processed to give three structural proteins (capsid (C), pre-membrane/membrane (prM/M), and envelope(E)) and seven nonstructural (NS) proteins (NS1, NS2A, NS2B, NS3, NS4A, NS4B, and NS5) [7]. These ZIKV proteins, especially NS2B-NS3 [8] and NS5 [9], have been actively explored as direct-acting antiviral targets in efforts to discover anti-ZIKV agents [10,11,12]. Furthermore, phenotypic screening of commercially available or in-house compound libraries has also been pursued as an alternative strategy [13]; however, identifying the molecular target of screening hits remains a challenging task and further hit-to-lead optimization requires significant medicinal chemistry efforts. Moreover, repurposing advanced small molecules, especially those that have been fully evaluated in clinical trials, is an effective approach to identifying ZIKV antivirals [14,15,16].

**Figure 1:**
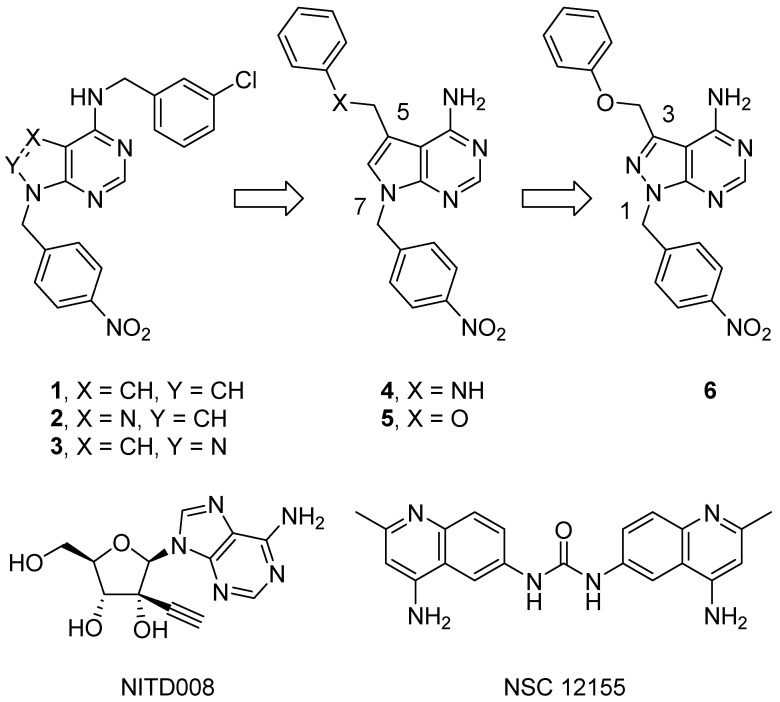
Selected known ZIKV inhibitors.

In our initial efforts to identify anti-ZIKV agents, we have discovered several inhibitors including compound **1** (Figure 1) that display promising anti-ZIKV activity, especially in the gold standard titer-reduction assay [17]. We have also demonstrated that compound **1**’s 7*H*-pyrrolo[2,3-*d*]pyrimidine scaffold can be replaced by 9*H*-purine or 1*H*-pyrazolo[3,4-*d*]pyrimidine as shown in compounds **2** and **3**, respectively. These compounds feature a substitution pattern as exemplified by the one in 4,7-disubstituted 7*H*-pyrrolo[2,3-*d*]pyrimidine **1**. To search for new anti-ZIKV chemotypes, we have examined alternative substitution patterns built on previously reported 7*H*-pyrrolo[2,3-*d*]pyrimidine and 1*H*-pyrazolo[3,4-*d*]pyrimidine central scaffolds. Here we report the structure-activity relationship (SAR) studies and highlight structural features that contribute to antiviral activity.

## 2. Results and Discussion

In the current study, we continued to use our previously reported luciferase-based ZIKV reporter assay as a primary antiviral screening, by which newly synthesized compounds were tested (at 10 µM) along with NITD008 (Figure 1, at 1 µM) [18], a flavivirus RNA-dependent RNA polymerase (RdRp) inhibitor, and NSC 12155 (Figure 1, at 10 µM) [19], a flavivirus NS5 methyltransferase (MTase) inhibitor, as reference compounds (Table 1). Inhibitors that showed good anti-ZIKV activity and relatively low cytotoxicity in the reporter assay were tested in the gold standard titer reduction assay at 8.5 µM. For active compounds (>60% titer-reduction), dose-response experiments were performed to determine EC_50_ (half maximal effective concentration, a concentration that is needed to cause half of the maximum possible pharmacological effect) and CC_50_ (half cytotoxic concentration, a concentration that is required to reduce cell viability by 50%) values. For selected compounds, EC_50_ values in the reporter assay were also measured.

We first examined compounds **4** and **5**, in which a 7*H*-pyrrolo[2,3-*d*]pyrimidine core structure was retained with an altered substitution pattern (Figure 1). In both compounds, a substituent was placed at position 5 in contrast to position 4 in compound **1**. Furthermore, instead of the benzyl amine functionality in compound **1**, a methylamino and methoxy linkage was used in compounds **4** and **5**, respectively. When tested at 10 µM in the reporter assay, compound **4** showed excellent antiviral activity (Table 1), which unfortunately could be due to its high intrinsic toxicity. In comparison, compound **5** was significantly less toxic and possessed good anti-ZIKV activity in the reporter assay. Compound **5**’s excellent antiviral activity (94% reduction at 8.5 µM) was revealed in the titer reduction assay and its EC_50_ (titer reduction) and CC_50_ values were further determined as 4.3 µM and 58 µM, respectively (Table 1). Given the fact that compound **1** had EC_50_ and CC_50_ values of 5.2 µM and 20 µM, respectively, compound **5** possessed comparable antiviral activity and was significantly less toxic. These results suggested that compound **5** represented a new, promising substitution pattern built on the 7*H*-pyrrolo[2,3-*d*]pyrimidine scaffold and warranted further SAR studies.

**Scheme 1:**
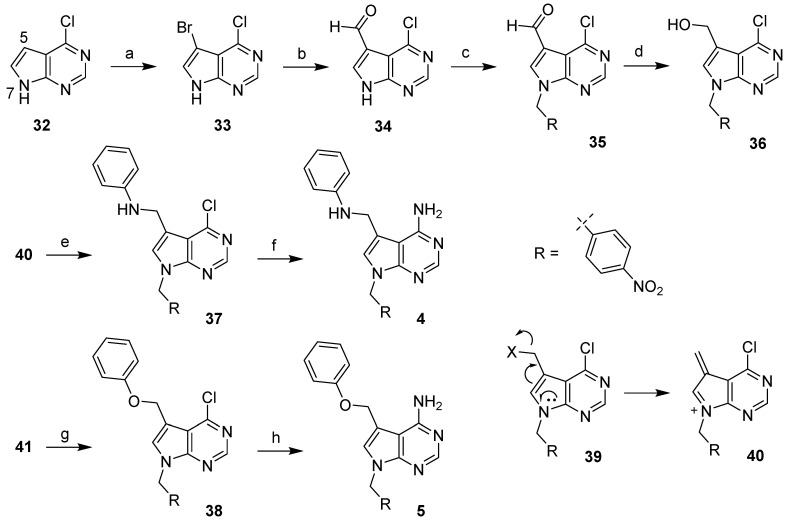
Synthesis of 5,7-disubstituted 7*H*-pyrrolo[2,3-*d*]pyrimidin-4-amine analogs. Reagents and conditions: (a) NBS, DMF; (b) ^n^BuLi, –78 °C, THF/hexanes; then DMF, rt; (c) 4-nitrobenzyl bromide, Cs_2_CO_3_, CH_3_CN; (d) NaBH_4_, MeOH/THF, 0 °C; (e) aniline, Na(OAc)_3_BH, AcOH, dichloroethane; (f) ammonia solution, dioxane, 120 °C; (g) phenol, Ph_3_P, DEAD, THF; (h) ammonia solution, dioxane, 120 °C.

Unfortunately, the synthesis of compound **5** proved to be extremely difficult most likely due to an intrinsic elimination reaction of activated intermediate **39** (see discussion below and Scheme 1). To facilitate SAR studies, we chose to investigate compound **6** and its analogs (Figure 1), which were based on 1*H*-pyrazolo[3,4-*d*]pyrimidine, a chemical type that was expected to circumvent the elimination reaction encountered during the synthesis of compound **5**. Compound **6** also had a substitution pattern identical to that of compound **5**, featuring two substituents at positions 1 and 3 of 1*H*-pyrazolo[3,4-*d*]pyrimidine, respectively (Figure 1). When tested in the reporter virus assay, compound **6** displayed anti-ZIKV and cytotoxicity at levels almost identical to those of compound **5** (Table 2). Moreover, it had EC_50_ (titer reduction) and CC_50_ values of 5.1 µM and 39 µM, respectively. The fact that compounds **5** and **6** possessed similar antiviral activity and cytotoxicity in both assays clearly indicated that 1*H*-pyrazolo[3,4-*d*]pyrimidine could indeed serve as an alternative scaffold. Therefore, we decided to focus on compound **6** analogs, which were synthetically more accessible and proceeded to explore the substituents at positions 1 and 3.

At position 1, compound **6** contained a phenyl ring (ring A), where a nitro group was attached at the para position. As our initial efforts, we prepared compound **7** with no substituents on ring A (Table 2). While compound **7** was not toxic, its anti-ZIKV activity was also abolished. This result suggested that a substituent at the para position was crucial to anti-ZIKV activity. To examine the effect of the modification site, we prepared compounds **8** and **9**, which had a nitro group at the ortho and meta position, respectively. Like compound **7**, compound **8** also lost its anti-ZIKV activity, eliminating the ortho position as a viable modification site. On the other hand, while compound **9** exhibited enhanced anti-ZIKV activity, its high cytotoxicity suggested that the meta position was not an ideal modification site. Since the nitro group was an electron-withdrawing group, we proceeded to prepare compounds **10–12**, in which the nitro group was reduced to the corresponding electron-donating aniline group (Table 2). None of these compounds displayed observable anti-ZIKV activity in the reporter assay, suggesting an electron-withdrawing group was preferred for antiviral activity. Taken together, these results demonstrated that an electron-withdrawing group at the para position of ring A was highly desirable.

Accordingly, we continued to investigate additional electron-withdrawing groups by preparing compounds **13–15**, in which trifluoromethyl, methylsulfonyl, and cyano, respectively, were placed at the para position of ring A (Table 2). Compound **13** showed anti-ZIKV activity and cytotoxicity at levels very similar to those of compound **6** in the reporter assay, suggesting that trifluoromethyl is also a productive substituent. This finding was further supported by compound **13**’s good antiviral activity (EC_50_ = 6.5 µM) in the titer reduction assay. In comparison, compounds **14** and **15** exhibited relatively lower anti-ZIKV activity. Nonetheless, their significant anti-ZIKV activity confirmed the beneficial effect of a para-positioned electron-withdrawing group when compared with non-substituted compound **7**. Taken together, these results showed that other electron-withdrawing groups such as trifluoromethyl could be used to enhance antiviral activity.

Encouraged by the above finding, we decided to explore methyl ester (Table 2), a functional group that was electron-withdrawing and, if necessary, could be further converted into other derivatives. When a methyl ester was placed at the ortho position, the resulting compound **16** had no anti-ZIKV activity in the reporter assay, indicating that modifications at this position had a detrimental effect. Compound **17**, which contained a meta-positioned methyl ester, had excellent anti-ZIKV activity; however, such activity was accompanied by high cytotoxicity. These SAR trends were in line with those observed when a nitro group was examined in compounds **8** and **9**. Surprisingly, compound **18**, in which a methyl ester was placed at the para position, had minimal anti-ZIKV activity, a SAR trend that clearly contradicted that seen in compounds **6** vs. **7**. This finding suggested that an increased steric effect imposed by a methyl ester group was not tolerated at the para position. To examine the steric effect, methyl ester **18** was converted into the corresponding primary amide **19**, a transformation that restored anti-ZIKV activity and cytotoxicity to levels similar to those of compound **6**. Unfortunately, compound **19** exhibited low antiviral activity in the titer reduction assay, an observation for further investigation in the future.

We next investigated whether the phenyl ring in compound **6** and its analogs could be replaced by a pyridine ring by taking advantage of the latter’s electron-deficient nature (Table 2). We first synthesized compounds **20** and **21**, in which an electron-withdrawing chloro group was attached at the meta and para position, respectively. Both compounds showed good anti-ZIKV activity in the reporter assay even though compound **21** was less toxic than **20**. The finding generally mirrored the SAR trend observed in compounds **6** vs. **9**. When tested in the titer reduction assay, while compound **21** possessed antiviral activity (EC_50_ = 5.2 µM) almost identical to that of compound **6**, it showed enhanced cytotoxicity (CC_50_ = 25 µM). We also explored the electron-donating amino group, which was placed at the meta and para position, leading to compounds **22** and **23**, respectively. Both compounds still retained significant anti-ZIKV activity. This finding contradicted the one seen in compounds **11** and **12**, where an amino group abolished anti-ZIKV activity. We also evaluated synthetic intermediates **24** and **25**, which displayed no drastic change in anti-ZIKV activity or cytotoxicity in comparison with compounds **22** and **23**, respectively. Taken together, these results suggested that a pyridine ring could be used to replace the phenyl ring in compound **6**; however, such isosteric change might lead to different SAR trends, which warrant future investigation.

After examining ring A, we continued to investigate the effect of substitution on ring B (Table 3). To that end, a para-NO_2_ substituted phenyl ring A was retained and an electron-donating methyl group was introduced at different positions of ring B, giving rise to compounds **26–28**. These compounds displayed anti-ZIKV activity and cytotoxicity comparable to those of compound **6**, suggesting that an electron-donating methyl group exerted minimal effect regardless of the substitution pattern. When an electron-withdrawing chloro group was attached at different positions of ring B, the resulting compounds **29–31** exhibited lower anti-ZIKV activity in comparison with compound **6**, pointing to chloro group’s having a negative effect. Taken together, these findings suggested that ring B was less sensitive to structural modifications. Given that methyl and chloro groups were relatively simple, substituents or functional groups that would elicit additional interactions, such as hydrogen bonds, could be candidates for future SAR investigation of ring B.

The syntheses of 5,7-disubstituted 7*H*-pyrrolo[2,3-*d*]pyrimidin-4-amines **4** and **5** were depicted in Scheme 1. Bromination of commercially available 4-chloro-7*H*-pyrrolo[2,3-*d*]pyrimidine (**32**) was performed by following literature procedures to give bromide **33** [20]. The introduction of a formyl group at position 5 was accomplished by halogen-metal exchange at −78 °C followed by treatment with dimethylformamide (DMF), giving rise to aldehyde **34** in excellent yields. Subsequent alkylation with 4-nitrobenzyl bromide gave aldehyde **35**, which was reduced to the corresponding alcohol **36**. Reductive amination reaction of aldehyde intermediate **35** and aniline afforded chloride **37**, which was subsequently subjected to aminolysis at high temperature to give compound **4** in 18% yield. To prepare compound **5**, alcohol intermediate **36** was initially activated through the formation of a chloride or mesylate followed by a nucleophilic attack; Unfortunately, these synthetic methods failed most likely because of an activated intermediate (generally represented by **39**) that readily underwent elimination reaction to generate an unstable, unproductive intermediate **40**. Alternatively, the Mitsunobu reaction of alcohol **36** and phenol gave rise to a complex reaction mixture most likely due to an oxyphosphonium intermediate **39**, which was also susceptible to elimination reaction. Nonetheless, when the mixture was subjected to aminolysis without further purification, compound **5** was obtained with a very low combined yield.

**Scheme 2:**
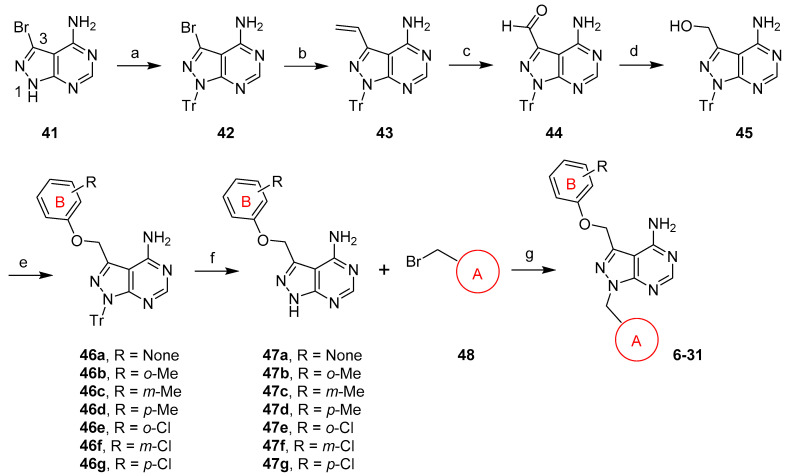
Synthesis of 1,3-disubstituted 1*H*-pyrazolo[3,4-*d*]pyrimidin-4-amine analogs. Reagents and conditions: (a) TrCl, Cs_2_CO_3_, DMF, 50 °C; (b) Bu_3_Sn(CH=CH_2_), Pd(PPh_3_)_4_, toluene, 110 °C; (c) OsO_4_, NaIO_4_, dioxane/H_2_O, rt; (d) NaBH_4_, MeOH/THF, 0 °C; (e) i. MsCl, Et_3_N, DMAP, CH_2_Cl_2_; ii. varied phenols, Cs_2_CO_3_, DMF; (f) TFA, Et_3_SiH, CH_2_Cl_2_, 0 °C; (g) Cs_2_CO_3_, CH_3_CN or DMF.

Since the synthesis of compound **5** was extremely difficult, we focused on compounds featuring a 1*H*-pyrazolo[3,4-*d*]pyrimidine core structure, whose preparation was depicted in Scheme 2. Commercially available 3-bromo-1*H*-pyrazolo[3,4-*d*]pyrimidin-4-amine (**41**) was first protected on the *N1* position to give trityl **42**, which was further modified on position 3. After the Stille coupling reaction, bromide **42** was converted into olefin **43**, whose double bond was cleaved to form aldehyde **44** in excellent yields. Reduction of **44** gave alcohol **45**, which was in turn used to prepare the key intermediate **46**. Gratifyingly, when alcohol **45** was activated via a mesylate and subsequently replaced with different phenols, phenyl ether **46** was obtained in moderate to good yields without major side products. After the trityl group in **46** was removed, the resulting free *N1* in **47** was alkylated with bromide **48** followed by additional transformations, if needed, to give compound **6–31** in a range of yields.

## 3. Conclusions

Expanding on our 7*H*-pyrrolo[2,3-*d*]pyrimidines ZIKV inhibitors, we have demonstrated that the central 7*H*-pyrrolo[2,3-*d*]pyrimidine scaffold can be donned with a different substitution pattern, giving rise to compound **5** as a low micromolar anti-ZIKV inhibitor. To circumvent the synthetic difficulties associated with compound **5**, we have designed compounds based on a 1*H*-pyrazolo[3,4-*d*]pyrimidine scaffold as exemplified by compound **6**. SAR studies of these compounds have revealed that an electron-withdrawing group at the para position of ring A is preferred for enhanced antiviral activity. Furthermore, it is feasible to replace the phenyl ring A in compound **6** with a pyridine ring without sacrificing antiviral activity. Nonetheless, we have also shown that relatively simple structural modifications on ring B have a minor impact on anti-ZIKV activity and cytotoxicity. Overall, we have discovered an alternative substitution pattern built on a 1*H*-pyrazolo[3,4-*d*]pyrimidine scaffold, leading to anti-ZIKV compounds including **6** and **13**. These new compounds together with those based on 7*H*-pyrrolo[2,3-*d*]pyrimidines, 9*H*-purines, and 1*H*-pyrazolo[3,4-*d*]pyrimidines represent promising starting chemotypes to discover new anti-ZIKV agents.

## 4. Experimental Section

### 4.1. General Procedures

All commercial reagents were used as provided unless otherwise indicated. An anhydrous solvent dispensing system (J.C. Meyer) using 2 packed columns of neutral alumina was used for drying THF, Et_2_O, and CH_2_Cl_2,_ whereas 2 packed columns of molecular sieves were used to dry DMF. Solvents were dispensed under argon. Flash chromatography was performed with RediSep R_f_ silica gel columns on a Teledyne ISCO CombiFlash^®^ R_f_ system using the solvents as indicated. Nuclear magnetic resonance spectra were recorded on a Varian 600 MHz or Bruker 400 MHz spectrometer with Me_4_Si or signals from the residual solvent as the internal standard for ^1^H or ^13^C. Chemical shifts are reported in ppm, and signals are described as s (singlet), d (doublet), t (triplet), q (quartet), m (multiplet), br s (broad singlet), and dd (double doublet). Values given for coupling constants are first order. High-resolution mass spectra were recorded on an Agilent TOF II TOF/MS instrument equipped with either an ESI or APCI interface at the Center for Drug Design, University of Minnesota.

### 4.2. Chemistry

#### 4.2.1. 5-Bromo-4-chloro-7H-pyrrolo[2,3-d]pyrimidine (**33**) [20]

To a solution of 4-chloro-7*H*-pyrrolo[2,3-*d*]pyrimidine (**32**, 2.51 g, 16.3 mmol) in anhydrous DMF (50 mL) was added NBS (2.91 g, 16.4 mmol) in anhydrous DMF (22 mL) in 25 min. The resulting mixture was stirred at rt for 1 h and poured into vigorously stirring water (350 mL). The precipitate was filtered, washed with water, suction-dried, and dried *in vacuo* to give compound **33** as a pale solid (3.55 g, 94%). ^1^H NMR (600 MHz, DMSO-*d_6_*) δ 13.00 (s, 1H), 8.63 (s, 1H), 7.96 (s, 1H). HRMS (ESI^−^) m/z calcd for C_6_H_2_BrClN_3_ [M-H]^−^ 229.9126, found 229.9124.

#### 4.2.2. 4-Chloro-7H-pyrrolo[2,3-d]pyrimidine-5-carbaldehyde (**34**)

^n^BuLi (1.6 M in hexanes, 10.5 mL, 16.8 mmol) was added dropwise to a solution of **33** (1.78 g, 7.66 mmol) in anhydrous THF (80 L) at −78 °C. The resulting mixture was stirred at −78 °C for 1 h, and anhydrous DMF (0.66 mL, 8.5 mmol) was added dropwise. After the reaction mixture was stirred at −78 °C for 30 min and then at room temperature overnight, it was cooled at 0 °C and carefully quenched with water (40 mL). The mixture was concentrated to produce a thick syrup, which was treated with saturated NH_4_Cl (60 mL) and vigorously stirred for 20 min. The solid that formed was filtered, washed with water (10 mL × 3), EtOAc (10 mL × 3), suction-dried, and dried *in vacuo* to give compound **34** as a pale solid (1.31 g, 94%). ^1^H NMR (600 MHz, DMSO-*d_6_*) δ 13.55 (s, 1H), 10.23 (s, 1H), 8.74 (s, 1H), 8.59 (s, 1H). HRMS (APCI^+^) m/z calcd for C_7_H_5_ClN_3_O [M + H]^+^ 182.0116, found 182.0109.

#### 4.2.3. 4-Chloro-7-(4-nitrobenzyl)-7H-nyrrolo[2,3-d]pyrimidine-5-carbaldehyde (**35**)

To a suspension of **34** (363 mg, 2.00 mmol) in anhydrous CH_3_CN (20 mL), K_2_CO_3_ (829 mg, 6.00 mmol) and 4-nitrobenzyl bromide (561 mg, 2.60 mmol) were added. The resulting mixture was stirred at room temperature for 23 h and then poured into stirring water (100 mL). The precipitate was filtered, washed with water, and suction-dried. The solid was suspended in ether (10 mL) and the mixture was vigorously stirred for 30 min. After filtration, the solid was washed with ether and dried *in vacuo* to give compound **35** as a light-yellow solid (576 mg, 91%). ^1^H NMR (600 MHz, CDCl_3_) δ 10.50 (s, 1H), 8.78 (s, 1H), 8.22 (d, *J* = 8.7 Hz, 2H), 8.06 (s, 1H), 7.45 (d, *J* = 8.7 Hz, 2H), 5.62 (s, 2H). HRMS (APCI^+^) m/z calcd for C_14_H_10_ClN_4_O_3_ [M + H]^+^ 317.0436, found 317.0440.

#### 4.2.4. 4-Chloro-7-(4-nitrobenzyl)-7H-pyrrolo[2,3-d]pyrimidin-5-yl)Methanol (**36**)

To a suspension of **35** (561 mg, 1.77 mmol) in THF (20 mL) and MeOH (20 mL) at 0 °C, NaBH_4_ (420 mg, 11.1 mmol) was added in small portions. After the resulting mixture was stirred at 0 °C for 30 min, the reaction was carefully quenched with an NH_4_Cl solution (30 mL of saturated NH_4_Cl and 10 mL of water), and the mixture was extracted with CHCl_3_ (40 × 2 mL). The combined organic layer was concentrated, and the residue was purified by flash column chromatography (0–10% MeOH/CH_2_Cl_2_) to give compound **36** as a yellow solid (473 mg, 84%). ^1^H NMR (600 MHz, CDCl_3_) δ 8.65 (s, 1H), 8.14 (d, *J* = 8.7 Hz, 2H), 7.37 (d, *J* = 8.7 Hz, 2H), 7.26 (s, 1H), 5.54 (s, 2H), 4.98 (s, 2H), 2.05 (s, 1H). HRMS (APCI^+^) m/z calcd for C_14_H_12_ClN_4_O_3_ [M + H]^+^ 319.0592, found 319.0587.

#### 4.2.5. N-((4-Chloro-7-(4-nitrobenzyl)-7H-pyrrolo[2,3-d]pyrimidin-5-yl)pethyl)pniline (**37**)

To a suspension of **35** (120 mg, 0.379 mmol) in anhydrous dichloroethane (6 mL), aniline (42 µL, 0.46 mmol), Na(OAc)_3_BH (241 mg, 1.14 mmol), and acetic acid (22 µL, 0.38 mmol) were added. The resulting mixture was stirred at room temperature for 40 h and diluted with CH_2_Cl_2_ (20 mL). The organic layer was washed with saturated NaHCO_3_ (10 mL), water (10 mL), and brine (20 mL). After concentration, the residue was purified by flash column chromatography (30–90% EtOAc/hexanes) to give compound **37** as a yellow solid (68 mg, 46%). ^1^H NMR (600 MHz, CDCl_3_) δ 8.64 (s, 1H), 8.15 (d, *J* = 8.7 Hz, 2H), 7.28 (d, *J* = 8.7 Hz, 2H), 7.19–7.16 (m, 3H), 6.74 (t, *J* = 7.2 Hz, 1H), 6.67–6.65 (m, 2H), 5.49 (s, 2H), 4.67 (s, 2H), 4.18 (s, 1H). HRMS (APCI^+^) m/z calcd for C_20_H_17_ClN_5_O_2_ [M + H]^+^ 394.1065, found 394.1060.

#### 4.2.6. 7-(4-Nitrobenzyl)-5-((phenylamino)methyl)-7H-pyrrolo[2,3-d]pyrimidin-4-amine (**4**)

A solution of **37** (61 mg, 0.16 mmol) in dioxane (3 mL) and strong ammonia solution (3 mL) was heated at 120 °C for 6 h in a sealed tube and allowed to cool to room temperature. The mixture was concentrated, and the residue was purified by flash column chromatography (0–10% MeOH/CH_2_Cl_2_) to give compound **4** as a yellow solid (11 mg, 18%). ^1^H NMR (600 MHz, CDCl_3_) δ 8.32 (s, 1H), 8.18 (d, *J* = 8.7 Hz, 2H), 7.34 (d, *J* = 8.7 Hz, 2H), 7.28–7.26 (m, 2H), 6.91–6.89 (m, 2H), 6.84–6.82 (m, 2H), 6.08 (s, 2H), 5.46 (s, 2H), 4.35 (s, 2H), 3.85 (s, 1H). HRMS (ESI^+^) m/z calcd for C_20_H_19_N_6_O_2_ [M + H]^+^ 375.1564, found 375.1561.

#### 4.2.7. 7-(4-Nitrobenzyl)-5-(phenoxymethyl)-7H-pyrrolo[2,3-d]pyrimidin-4-amine (**5**)

Phenol (58 mg, 0.62 mmol), Ph_3_P (162 mg, 0.618 mmol), and then DEAD (97 µL, 0.62 mmol, dropwise) were added to a solution of **36** (131 mg, 0.411 mmol) in anhydrous THF (7 mL) at 0 °C. The resulting mixture was stirred at 0 °C for 1 h and then at room temperature for 20 h. After concentration, the residue was purified by flash column chromatography (10–90% EtOAc/hexanes) to give a white solid (121 mg), which contained a small amount of compound **38**. The solid was dissolved in dioxane (5 mL) and strong ammonia solution (5 mL) in a sealed tube. The resulting mixture was heated at 120 °C for 6 h and allowed to cool to room temperature. After the mixture was concentrated, the residue was purified by preparative thin layer chromatography (5% MeOH/CH_2_Cl_2_) to give compound **5** as a yellowish solid (5 mg). ^1^H NMR (600 MHz, CDCl_3_/CD_3_OD) δ 8.17 (d, *J* = 8.4 Hz, 2H), 8.11 (s, 1H), 7.38–7.36 (m, 1H), 7.30 (d, *J* = 8.4 Hz, 2H), 7.10–7.04 (m, 2H), 6.90–6.84 (m, 2H), 6.80 (t, *J* = 8.4 Hz, 1H), 5.45 (s, 2H), 4.10 (s, 2H). HRMS (ESI^+^) m/z calcd for C_20_H_18_N_5_O_3_ [M + H]^+^ 376.1404, found 376.1400.

#### 4.2.8. 3-Bromo-1-trityl-1H-pyrazolo[3,4-d]pyrimidin-4-amine (**42**)

Cs_2_CO_3_ (6.55 g, 20.1 mmol) and trityl chloride (2.94 g, 10.5 mmol) were added to a solution of 3-bromo-1*H*-pyrazolo[3,4-*d*]pyrimidin-4-amine (**41**, 2.14 g, 10.0 mmol) in anhydrous DMF (50 mL). The resulting mixture was heated at 60 °C for 21 h, allowed to cool to room temperature, and poured into ice-water (300 mL). After the mixture was vigorously stirred for 30 min, the precipitate was filtered, washed with water, suction-dried, and dried *in vacuo* to give compound **42** as a white solid. (4.58 g, quantitative yield). ^1^H NMR (600 MHz, CDCl_3_) δ 8.05 (s, 1H), 7.32–7.21 (m, 15H), 5.91 (s, 2H). HRMS (ESI^+^) m/z calcd for C_24_H_19_BrN_5_ [M + H]^+^ 456.0818, found 456.0811.

#### 4.2.9. 1-Trityl-3-vinyl-1H-pyrazolo[3,4-d]pyrimidin-4-amine (**43**)

Pd(PPh_3_)_4_ (588 mg, 0.509 mmol) was added to a suspension of **42** (4.57 g, 10.0 mmol) in anhydrous toluene (100 mL). After the mixture was evacuated and backfilled with argon for three times, tributyl(vinyl)tin (3.51 mL, 12.0 mml) was added. The resulting mixture was heated at 110 °C for 6 h, allowed to cool to room temperature, diluted with EtOAc (200 mL), and treated with KF (7 g) for 15 min. After Celite was added, the mixture was stirred for 15 min and filtered. The filtrate was concentrated, and the residue was purified by flash column chromatography (20–100% EtOAc/hexanes) to give compound **43** as a yellowish solid (3.89 g, 96%). ^1^H NMR (600 MHz, CDCl_3_) δ 8.07 (s, 1H), 7.28–7.23 (m, 15H), 6.94 (dd, *J* = 17.8, 11.0 Hz, 1H), 5.90 (dd, *J* = 17.8, 1.3 Hz, 1H), 5.60–5.54 (m, 3H). HRMS (ESI^+^) m/z calcd for C_26_H_22_N_5_ [M + H]^+^ 404.1870, found 404.1867.

#### 4.2.10. 4-Amino-1-trityl-1H-pyrazolo[3,4-d]pyrimidine-3-carbaldehyde (**44**)

OsO_4_ solution (2.5 wt% in *^t^*BuOH, 0.94 mL, 0.075 mmol) was added to a suspension of **43** (3.00 g, 7.44 mmol) in dioxane (120 mL) and water (40 mL). After the mixture was stirred at room temperature for 30 min, NaIO_4_ (3.20 g, 15.0 mmol) was added. The resulting mixture was stirred for an additional 4 h, and dioxane was removed *in vacuo*. Water (60 mL) was added to the resulting slurry and the mixture was stirred for 15 min. The precipitate was filtered, washed with water, suction-dried, and dried *in vacuo* to give compound **44** as a pale solid (3.00 g, 99%). ^1^H NMR (600 MHz, CDCl_3_) δ 9.91 (s, 1H), 8.08 (s, 1H), 7.29–7.27 (m, 12H), 7.19–7.17 (m, 3H). HRMS (ESI^−^) m/z calcd for C_25_H_18_N_5_O [M-H]^−^ 404.1517, found 404.1515.

#### 4.2.11. 4-Amino-1-trityl-1H-pyrazolo[3,4-d]pyrimidin-3-yl)methanol (**45**)

NaBH_4_ (1.12 g, 29.6 mmol) was added in small portions to a solution of **44** (2.98 g, 7.35 mmol) in THF (50 mL) and MeOH (50 mL) at 0 °C. After the resulting mixture was stirred at 0 °C for 1 h, the reaction was carefully quenched with an NH_4_Cl solution (100 mL of saturated NH_4_Cl and 35 mL of water), and the mixture was extracted with CHCl_3_ (200 mL). The organic layer was dried over Na_2_SO_4_ and filtered. The filtrate was concentrated, and the residue was purified by flash column chromatography (0–10% MeOH/CH_2_Cl_2_) to give compound **45** as a white fluffy solid (2.42 g, 81%). ^1^H NMR (600 MHz, DMSO-*d_6_*) δ 7.86 (s, 1H), 7.27–7.13 (m, 15H), 6.20 (t, *J* = 5.3 Hz, 1H), 4.67 (d, *J* = 5.3 Hz, 2H). HRMS (ESI^+^) m/z calcd for C_25_H_22_N_5_O [M + H]^+^ 408.1819, found 408.1818.

#### 4.2.12. 3-(Phenoxymethyl)-1-trityl-1H-pyrazolo[3,4-d]pyrimidin-4-amine (**46a**)

Methanesulfonic anhydride (1.68 g, 9.64 mmol) was added to a suspension of **45** (3.29 g, 8.07 mmol), Et_3_N (1.35 mL, 9.68 mmol), and DMAP (100 mg, 0.82 mmol) in CH_2_Cl_2_ (80 mL) at 0 °C. The resulting mixture was stirred at 0 °C for 1 h and diluted with CHCl_3_ (240 mL). The organic layer was washed water (80 mL × 2) and brine (300 mL), and then concentrated to give a pale solid. The residue was immediately dissolved in anhydrous DMF (80 mL), then Cs_2_CO_3_ (7.90 g, 24.2 mmol) and phenol (1.60 g 17.0 mmol) were added. The resulting mixture was stirred at room temperature for 2 h, and DMF was removed *in vacuo*. The residue was partitioned between CHCl_3_ (300 mL) and water (100 mL). The organic layer was washed with water (100 mL) and brine (200 mL) and dried over Na_2_SO_4_. After filtration, the filtrate was concentrated, and the residue was purified by flash column chromatography (30–100% EtOAc/hexanes) to give compound **46a** as a pale solid (3.09 g, 79%). ^1^H NMR (600 MHz, CDCl_3_) δ 8.06 (s, 1H), 7.32–7.23 (m, 17H), 7.04–7.01 (m, 3H), 5.37 (s, 2H). No desired ionization observed in ESI or APCI modes.

#### 4.2.13. 3-((o-Tolyloxy)methyl)-1-trityl-1H-pyrazolo[3,4-d]pyrimidin-4-amine (**46b**)

Compound **46b** was prepared from **45** (540 mg, 1.33 mmol) and *o*-cresol (289 mg, 2.67 mmol) as described for compound **46a**. Pale solid, 447 mg, yield 68%. ^1^H NMR (600 MHz, CDCl_3_) δ 8.06 (s, 1H), 7.28–7.24 (m, 15H), 7.18–7.16 (m, 1H), 7.14 (td, *J* = 7.8, 1.4 Hz, 1H), 7.01–6.97 (m, 1H), 6.93 (td, *J* = 7.8, 1.2 Hz, 1H), 6.11 (s, 2H), 5.36 (s, 2H), 2.29 (s, 3H). No desired ionization was observed in ESI or APCI modes.

#### 4.2.14. 3-((m-Tolyloxy)methyl)-1-trityl-1H-pyrazolo[3,4-d]pyrimidin-4-amine (**46c**)

Compound **46c** was prepared from **45** (540 mg, 1.33 mmol) and *m*-cresol (289 mg, 2.67 mmol) as described for compound **46a**. Pale solid, 391 mg, yield 59%. ^1^H NMR (600 MHz, CDCl_3_) δ 8.06 (s, 1H), 7.28–7.23 (m, 15H), 7.19 (t, *J* = 7.8 Hz, 1H), 6.86–6.81 (m, 3H), 6.12 (s, 2H), 5.35 (s, 2H), 2.33 (s, 3H). No desired ionization was observed in ESI or APCI modes.

#### 4.2.15. 3-((p-Tolyloxy)methyl)-1-trityl-1H-pyrazolo[3,4-d]pyrimidin-4-amine (**46d**)

Compound **46d** was prepared from **45** (540 mg, 1.33 mmol) and *p*-cresol (289 mg, 2.67 mmol) as described for compound **46a**. White solid, 442 mg, yield 67%. ^1^H NMR (600 MHz, CDCl_3_) δ 8.05 (s, 1H), 7.28–7.24 (m, 15H), 7.10 (d, *J* = 8.5 Hz, 2H), 6.91 (d, *J* = 8.5 Hz, 2H), 6.13 (s, 2H), 5.33 (s, 2H), 2.31 (s, 3H). No desired ionization was observed in ESI or APCI modes.

#### 4.2.16. 3-((2-Chlorophenoxy)methyl)-1-trityl-1H-pyrazolo[3,4-d]pyrimidin-4-amine (**46e**)

Compound **46e** was prepared from **45** as described for compound **46a**. ^1^H NMR (600 MHz, CDCl_3_) δ 8.04 (s, 1H), 7.37 (dd, *J* = 7.9, 1.6 Hz, 1H), 7.25–7.21 (m, 15H), 7.16 (ddd, *J* = 8.9, 7.4, 1.6 Hz, 1H), 7.05 (dd, *J* = 8.4, 1.3 Hz, 1H), 6.94 (td, *J* = 7.7, 1.3 Hz, 1H), 6.13 (s, 2H), 5.44 (s, 2H). No desired ionization was observed in ESI or APCI modes.

#### 4.2.17. 3-((3-Chlorophenoxy)methyl)-1-trityl-1H-pyrazolo[3,4-d]pyrimidin-4-amine (**46f**)

Compound **46f** was prepared from **45** as described for compound **46a**. ^1^H NMR (600 MHz, CDCl_3_) δ 8.05 (s, 1H), 7.26–7.22 (m, 15H), 7.19 (t, *J* = 8.1 Hz, 1H), 7.05 (t, *J* = 2.2 Hz, 1H), 6.99 (dd, *J* = 8.3, 1.2 Hz, 1H), 6.88 (dd, *J* = 8.3, 2.5 Hz, 1H), 6.03 (s, 2H), 5.36(s, 2H). No desired ionization was observed in ESI or APCI modes.

#### 4.2.18. 3-((4-Chlorophenoxy)methyl)-1-trityl-1H-pyrazolo[3,4-d]pyrimidin-4-amine (**46g**)

Compound **46g** was prepared from **45** as described for compound **46a**. ^1^H NMR (600 MHz, CDCl_3_) δ 8.05 (s, 1H), 7.28–7.21 (m, 17H), 6.92 (dd, *J* = 8.9, 2.4 Hz, 2H), 6.26 (s, 2H), 5.33 (s, 2H). No desired ionization was observed in ESI or APCI modes.

#### 4.2.19. 3-(Phenoxymethyl)-1H-pyrazolo[3,4-d]pyrimidin-4-amine (**47a**)

Et_3_SiH (4.4 mL, 28 mmol) and then TFA (5.3 mL, 69 mmol) were added dropwise to a solution of **46a** (3.35 g, 6.93 mmol) in anhydrous CH_2_Cl_2_ (140 mL) at 0 °C. The resulting mixture was stirred at 0 °C for 30 min and then at room temperature for 2 h. After concentration, the residue was co-evaporated with toluene (100 mL × 2), and the resulting solid was triturated with warm hexanes (100 mL) and saturated NaHCO_3_ (20 mL). The mixture was filtered and the solid was washed with hexanes (25 mL × 2) and water (10 mL × 2), suction-dried, and dried *in vacuo* to give compound **47a** as a pale solid (1.61 g, 96%). ^1^H NMR (600 MHz, DMSO-*d_6_*) δ 13.56 (s, 1H), 8.21 (s, 1H), 7.35–7.27 (m, 2H), 7.07– 7.05 (m, 2H), 6.96 (t, *J* = 7.3 Hz, 1H), 5.43 (s, 2H). HRMS (ESI^+^) m/z calcd for C_12_H_12_N_5_O [M + H]^+^ 242.1036, found 242.1046.

#### 4.2.20. 3-((o-Tolyloxy)methyl)-1H-pyrazolo[3,4-d]pyrimidin-4-amine (**47b**)

Compound **47b** was prepared from **46b** (421 mg, 0.846 mmol) as described for compound **47a**. Pale solid, 190 mg, yield 88%. ^1^H NMR (600 MHz, DMSO-*d_6_*) δ 13.40 (s, 1H), 8.17 (s, 1H), 7.15–7.10 (m, 3H), 6.85 (t, *J* = 7.4 Hz, 1H), 5.42 (s, 2H), 2.15 (s, 3H). HRMS (ESI^+^) m/z calcd for C_13_H_14_N_5_O [M + H]^+^ 256.1193, found 256.1205.

#### 4.2.21. 3-((m-Tolyloxy)methyl)-1H-pyrazolo[3,4-d]pyrimidin-4-amine (**47c**)

Compound **47c** was prepared from **46c** (371 mg, 0.746 mmol) as described for compound **47a**. Pale solid, 179 mg, yield 94%. ^1^H NMR (600 MHz, DMSO-*d_6_*) δ 13.66 (s, 1H), 8.25 (s, 1H), 7.17 (t, *J* = 7.9 Hz, 1H), 6.89–6.78 (m, 3H), 5.41 (s, 2H), 2.27 (s, 3H). HRMS (ESI^+^) m/z calcd for C_13_H_14_N_5_O [M + H]^+^ 256.1193, found 256.1192.

#### 4.2.22. 3-((p-tolyloxy)methyl)-1H-pyrazolo[3,4-d]pyrimidin-4-amine (**47d**)

Compound **47d** was prepared from **46d** (417 mg, 0.838 mmol) as described for compound **47a**. White solid, 196 mg, yield 92%. ^1^H NMR (600 MHz, DMSO-*d_6_*) δ 13.65 (s, 1H), 8.24 (s, 1H), 7.09 (d, *J* = 8.1 Hz, 2H), 6.94 (d, *J* = 8.1 Hz, 2H), 5.40 (s, 2H), 2.22 (s, 3H). HRMS (ESI^+^) m/z calcd for C_13_H_14_N_5_O [M + H]^+^ 256.1193, found 256.1201.

#### 4.2.23. 3-((2-Chlorophenoxy)methyl)-1H-pyrazolo[3,4-d]pyrimidin-4-amine (**47e**)

Compound **47e** was prepared from **46e** as described for compound **47a**. ^1^H NMR (600 MHz, CDCl_3_) δ 10.80 (s, 1H), 8.34 (s, 1H), 7.41 (dd, *J* = 8.0, 1.2 Hz, 1H), 7.22 (td, *J* = 8.0, 1.2 Hz, 1H), 7.11 (d, *J* = 8.0 Hz, 1H), 6.99 (t, *J* = 7.8 Hz, 1H), 5.52 (s, 2H). HRMS (ESI^+^) m/z calcd for C_12_H_11_ClN_5_O [M + H]^+^ 276.0647, found 276.0637.

#### 4.2.24. 3-((3-Chlorophenoxy)methyl)-1H-pyrazolo[3,4-d]pyrimidin-4-amine (**47f**)

Compound **47f** was prepared from **46f** as described for compound **47a**. ^1^H NMR (600 MHz, CDCl_3_) δ 10.54 (s, 1H), 8.39 (s, 1H), 7.23 (t, *J* = 8.1 Hz, 1H), 7.06 (t, *J* = 2.1 Hz, 1H), 7.01 (ddd, *J* = 8.2, 2.1, 0.8 Hz, 1H), 6.94 (ddd, *J* = 8.1, 2.1, 0.8 Hz, 1H), 6.05 (s, 2H), 5.43 (s, 2H). HRMS (ESI^+^) m/z calcd for C_12_H_11_ClN_5_O [M + H]^+^ 276.0647, found 276.0647.

#### 4.2.25. 3-((4-Chlorophenoxy)methyl)-1H-pyrazolo[3,4-d]pyrimidin-4-amine (**47g**)

Compound **47g** was prepared from **46g** as described for compound **47a**. ^1^H NMR (600 MHz, CDCl_3_) δ 10.63 (s, 1H), 8.39 (s, 1H), 7.26 (d, *J* = 9.0 Hz, 2H), 6.98 (d, *J* = 9.0 Hz, 2H), 5.42 (s, 2H). HRMS (ESI^+^) m/z calcd for C_12_H_11_ClN_5_O [M + H]^+^ 276.0647, found 276.0657.

#### 4.2.26. 1-(4-Nitrobenzyl)-3-(phenoxymethyl)-1H-pyrazolo[3,4-d]pyrimidin-4-amine (**6**)

Cs_2_CO_3_ (216 mg, 0.663 mmol) and 4-nitrobenzyl bromide (88 mg, 0.41 mmol) were added to a suspension of **47a** (80 mg, 0.33 mmol) in anhydrous CH_3_CN (4 mL). The resulting mixture was stirred at room temperature for 6 h and concentrated, then the residue was then dissolved in EtOAc (20 mL). The organic layer was washed with water (10 mL × 3) and brine (20 mL) and dried over Na_2_SO_4_. After filtration, the filtrate was concentrated, and the residue was purified by flash column chromatography (0–5% MeOH/CH_2_Cl_2_) to give compound **6** as a pale solid (70 mg, 56%). ^1^H NMR (600 MHz, CDCl_3_) δ 8.36 (s, 1H), 8.16 (d, *J* = 8.4 Hz, 2H), 7.41 (d, *J* = 8.4 Hz, 2H), 7.32– 7.29 (m, 2H), 7.05–7.01 (m, 3H), 6.27 (s, 2H), 5.64 (s, 2H), 5.40 (s, 2H). HRMS (ESI^+^) m/z calcd for C_19_H_17_N_6_O_3_ [M + H]^+^ 377.1357, found 377.1360.

#### 4.2.27. 1-Benzyl-3-(phenoxymethyl)-1H-pyrazolo[3,4-d]pyrimidin-4-amine (**7**)

Compound **7** was prepared from **47a** (49 mg, 0.20 mmol) and benzyl bromide (32 µL, 0.27 mmol) as described for compound **6**. White solid, 9 mg, yield 13%. ^1^H NMR (600 MHz, CDCl_3_) δ 8.37 (s, 1H), 7.31–7.28 (m, 7H), 7.03–7.01 (m, 3H), 6.22 (s, 2H), 5.55 (s, 2H), 5.40 (s, 2H). HRMS (ESI^+^) m/z calcd for C_19_H_18_N_5_O [M + H]^+^ 332.1506, found 332.1504.

#### 4.2.28. 1-(2-Nitrobenzyl)-3-(phenoxymethyl)-1H-pyrazolo[3,4-d]pyrimidin-4-amine (**8**)

Compound **8** was prepared from **47a** (100 mg, 0.414 mmol) and 2-nitrobenzyl bromide (107 mg, 0.495 mmol) as described for compound **6**. White solid, 84 mg, yield 54%. ^1^H NMR (600 MHz, CDCl_3_) δ 8.35 (s, 1H), 8.17–8.16 (m, 1H), 7.46–7.43 (m, 2H), 7.33–7.30 (m, 2H), 7.05–7.02 (m, 3H), 6.62–6.60 (m, 1H), 6.15 (s, 2H), 6.03 (s, 2H), 5.43 (s, 2H). HRMS (ESI^+^) m/z calcd for C_19_H_17_N_6_O_3_ [M + H]^+^ 377.1357, found 377.1361.

#### 4.2.29. 1-(3-Nitrobenzyl)-3-(phenoxymethyl)-1H-pyrazolo[3,4-d]pyrimidin-4-amine (**9**)

Compound **9** was prepared from **47a** (80 mg, 0.33 mmol) and 3-nitrobenzyl bromide (88 mg, 0.41 mmol) as described for compound **6**. White solid, 63 mg, yield 50%. ^1^H NMR (600 MHz, CDCl_3_) δ 8.37 (s, 1H), 8.20 (t, *J* = 2.0 Hz, 1H), 8.15 (ddd, *J* = 8.2, 2.0, 1.1 Hz, 1H), 7.62 (ddd, *J* = 7.8, 1.8, 1.1 Hz, 1H), 7.49 (t, *J* = 7.9 Hz, 1H), 7.32–7.29 (m, 2H), 7.04–7.01 (m, 3H), 6.21 (s, 2H), 5.64 (s, 2H), 5.41 (s, 2H). HRMS (ESI^+^) m/z calcd for C_19_H_17_N_6_O_3_ [M + H]^+^ 377.1357, found 377.1357.

#### 4.2.30. 1-(2-Aminobenzyl)-3-(phenoxymethyl)-1H-pyrazolo[3,4-d]pyrimidin-4-amine (**10**)

SnCl_2_ (198 mg, 1.04 mmol) was added to a suspension of **8** (56 mg, 0.15 mmol) in EtOH (5 mL). The resulting mixture was heated at 70 °C for 18 h, allowed to cool to room temperature, and diluted with EtOAc (12 mL). The reaction was carefully quenched with a NaHCO_3_ solution (6 mL of saturated NaHCO_3_ and 1.5 mL of water). The mixture was filtered through a pad of Celite and washed with 5% MeOH/EtOAc. The filtrate was separated, and the organic layer was washed with brine (20 mL × 2) and dried over Na_2_SO_4_. After filtration, the filtrate was concentrated, and the residue was purified by flash column chromatography (0–10% MeOH/CH_2_Cl_2_) to give compound **10** as a pale solid (41 mg, 79%). ^1^H NMR (600 MHz, DMSO-*d_6_*) δ 8.24 (s, 1H), 7.84 (s, 2H), 7.31–7.28 (m, 2H), 7.06–7.04 (m, 2H), 6.99–6.95 (m, 2H), 6.79 (dd, *J* = 7.6, 1.6 Hz, 1H), 6.65 (dd, *J* = 8.0, 1.2 Hz, 1H), 6.47 (td, *J* = 7.6, 1.2 Hz, 1H), 5.42 (s, 2H), 5.31 (s, 2H), 5.29 (s, 2H). HRMS (ESI^+^) m/z calcd for C_19_H_19_N_6_O [M + H]^+^ 347.1615, found 347.1615.

#### 4.2.31. 1-(3-Aminobenzyl)-3-(phenoxymethyl)-1H-pyrazolo[3,4-d]pyrimidin-4-amine (**11**)

Compound **11** was prepared from **9** (48 mg, 0.13 mmol) as described for compound **10**. White solid, 34 mg, yield 77%. ^1^H NMR (600 MHz, DMSO-*d_6_*) δ 8.23 (s, 1H), 7.75 (s, 2H), 7.31–7.28 (m, 2H), 7.06–7.04 (m, 2H), 6.96 (td, *J* = 7.3, 1.1 Hz, 1H), 6.92 (t, *J* = 7.7 Hz, 1H), 6.43 (ddd, *J* = 8.0, 2.2, 1.2 Hz, 1H), 6.40 (s, 1H), 6.36 (dd, *J* = 7.7, 1.2 Hz, 1H), 5.41 (s, 2H), 5.31 (s, 2H), 5.06 (s, 2H). HRMS (ESI^+^) m/z calcd for C_19_H_19_N_6_O [M + H]^+^ 347.1615, found 347.1620.

#### 4.2.32. 1-(4-Aminobenzyl)-3-(phenoxymethyl)-1H-pyrazolo[3,4-d]pyrimidin-4-pmine (**12**)

Compound **12** was prepared from **6** (58 mg, 0.15 mmol) as described for compound **10**. Pale solid, 37 mg, yield 69%. ^1^H NMR (600 MHz, DMSO-*d_6_*) δ 8.22 (s, 1H), 7.83 (s, 2H), 7.30–7.27 (m, 2H), 7.04 (d, *J* = 8.4 Hz, 2H), 6.97–6.94 (m, 3H), 6.46 (d, *J* = 8.4 Hz, 2H), 5.40 (s, 2H), 5.27 (s, 2H), 5.04 (2H). HRMS (ESI^+^) m/z calcd for C_19_H_19_N_6_O [M + H]^+^ 347.1615, found 347.1616.

#### 4.2.33. 3-(Phenoxymethyl)-1-(4-(trifluoromethyl)benzyl)-1H-pyrazolo[3,4-d]pyrimidin-4-amine (**13**)

Compound **13** was prepared from **47a** (40 mg, 0.17 mmol) and 4-(trifluoromethyl)benzyl bromide (50 mg, 0.21 mmol) as described for compound **6**. White solid, 32 mg, yield 48%. ^1^H NMR (600 MHz, CDCl_3_) δ 8.37 (s, 1H), 7.56 (d, *J* = 8.0 Hz, 2H), 7.39 (d, *J* = 8.0 Hz, 2H), 7.31–7.28 (m, 2H), 7.04–7.01 (m, 3H), 6.22 (s, 2H), 5.60 (s, 2H), 5.40 (s, 2H). HRMS (ESI^+^) m/z calcd for C_20_H_17_F_3_N_5_O [M + H]^+^ 400.1380, found 400.1375.

#### 4.2.34. 1-(4-(Methylsulfonyl)Benzyl)-3-(phenoxymethyl)-1H-pyrazolo[3,4-d]pyrimidin-4-amine (**14**)

Compound **14** was prepared from **47a** (40 mg, 0.17 mmol) and 4-(methylsulfonyl)benzyl bromide (52 mg, 0.21 mmol) as described for compound **6**. White solid, 23 mg, yield 34%. ^1^H NMR (600 MHz, CDCl_3_) δ 8.36 (s, 1H), 7.88 (d, *J* = 8.4 Hz, 2H), 7.46 (d, *J* = 8.4 Hz, 2H), 7.32–7.29 (m, 2H), 7.04–7.01 (m, 3H), 6.25 (s, 2H), 5.63 (s, 2H), 5.40 (s, 2H), 3.01 (s, 3H). HRMS (ESI^+^) m/z calcd for C_20_H_20_N_5_O_3_S [M + H]^+^ 410.1281, found 410.1289.

#### 4.2.35. 4-((4-Amino-3-(phenoxymethyl)-1H-pyrazolo[3,4-d]pyrimidin-1-yl)methyl)benzonitrile (**15**)

Compound **15** was prepared from **47a** (40 mg, 0.17 mmol) and 4-cyanobenzyl bromide (39 mg, 0.20 mmol) as described for compound **6**. White solid, 34 mg, yield 57%. ^1^H NMR (600 MHz, CDCl_3_) δ 8.35 (s, 1H), 7.60 (d, *J* = 8.2 Hz, 2H), 7.36 (d, *J* = 8.2 Hz, 2H), 7.31–7.29 (m, 2H), 7.04–7.01 (m, 3H), 6.26 (s, 2H), 5.59 (s, 2H), 5.39 (s, 2H). HRMS (ESI^+^) m/z calcd for C_20_H_17_N_6_O [M + H]^+^ 357.1458, found 357.1467.

#### 4.2.36. Methyl 2-((4-Amino-3-(phenoxymethyl)-1H-pyrazolo[3,4-d]pyrimidin-1-yl)methyl)benzoate (**16**)

Compound **16** was prepared from **47a** (100 mg, 0.41 mmol) and methyl 2-(bromomethyl)benzoate (115 mg, 0.50 mmol) as described for compound **6**. White solid, 41 mg, yield 25%. ^1^H NMR (600 MHz, CDCl_3_) δ 8.34 (s, 1H), 8.03 (dd, *J* = 7.2, 2.0 Hz, 1H), 7.35–7.29 (m, 4H), 7.05–7.01 (m, 3H), 6.53 (dd, *J* = 7.3, 1.7 Hz, 1H), 6.25 (s, 2H), 6.05 (s, 2H), 5.44 (s, 2H), 3.94 (s, 3H). HRMS (ESI^+^) m/z calcd for C_21_H_20_N_5_O_3_ [M + H]^+^ 390.1561, found 390.1567.

#### 4.2.37. Methyl 3-((4-Amino-3-(phenoxymethyl)-1H-pyrazolo[3,4-d]pyrimidin-1-yl)methyl)benzoate (**17**)

Compound **17** was prepared from **47a** (100 mg, 0.41 mmol) and methyl 3-(bromomethyl)benzoate (114 mg, 0.50 mmol) as described for compound **6**. White solid, 75 mg, yield 46%. ^1^H NMR (600 MHz, CDCl_3_) δ 8.37 (s, 1H), 8.03 (d, *J* = 1.9 Hz, 1H), 7.95 (dd, *J* = 7.7, 1.5 Hz, 1H), 7.46 (dd, *J* = 7.7, 1.6 Hz, 1H), 7.38 (t, *J* = 7.7 Hz, 1H), 7.31–7.27 (m, 2H), 7.03–7.01 (m, 3H), 6.22 (s, 2H), 5.59 (s, 2H), 5.39 (s, 2H), 3.89 (s, 3H). HRMS (ESI^+^) m/z calcd for C_21_H_20_N_5_O_3_ [M + H]^+^ 390.1561, found 390.1562.

#### 4.2.38. Methyl 4-((4-amino-3-(phenoxymethyl)-1H-pyrazolo[3,4-d]pyrimidin-1-yl)methyl)benzoate (**18**)

Compound **18** was prepared from **47a** (100 mg, 0.41 mmol) and methyl 4-(bromomethyl)benzoate (115 mg, 0.50 mmol) as described for compound **6**. White solid, 80 mg, yield 50%. ^1^H NMR (600 MHz, CDCl_3_) δ 8.36 (s, 1H), 7.97 (d, *J* = 8.3 Hz, 2H), 7.32–7.28 (m, 4H), 7.03–7.01 (m, 3H), 6.23 (s, 2H), 5.60 (s, 2H), 5.40 (s, 2H), 3.89 (s, 3H). HRMS (ESI^+^) m/z calcd for C_21_H_20_N_5_O_3_ [M + H]^+^ 390.1561, found 390.1566.

#### 4.2.39. 4-((4-Amino-3-(phenoxymethyl)-1H-pyrazolo[3,4-d]pyrimidin-1-yl)methyl)benzamide (**19**)

A mixture of **18** (50 mg, 0.13 mmol), CaCl_2_ (55 mg, 0.50 mmol), and 7 N NH_3_/MeOH (8 mL) was heated at 90 °C for 24 h in a sealed tube and then allowed to cool to room temperature. The mixture was concentrated, and the residue was treated with 10% MeOH/CH_2_Cl_2_ (20 mL). The organic phase was concentrated, and the residue was purified by flash column chromatography (0–10% MeOH/CH_2_Cl_2_) to give compound **19** as a white solid (37 mg, 77%). ^1^H NMR (600 MHz, DMSO-*d_6_*) δ 8.23 (s, 1H), 7.92 (s, 1H), 7.79 (d, *J* = 8.4 Hz, 2H), 7.34 (s, 1H), 7.32–7.28 (m, 2H), 7.24 (d, *J* = 8.4 Hz, 2H), 7.04 (d, *J* = 8.4 Hz, 2H), 6.96 (t, *J* = 7.5 Hz, 1H), 5.55 (s, 2H), 5.43 (s, 2H). HRMS (ESI^+^) m/z calcd for C_20_H_19_N_6_O_2_ [M + H]^+^ 375.1564, found 375.1558.

#### 4.2.40. 1-((2-Chloropyridin-4-yl)methyl)-3-(phenoxymethyl)-1H-pyrazolo[3,4-d]pyrimidin-4-amine (**20**)

Compound **20** was prepared from **47a** (80 mg, 0.33 mmol) and 4-(bromomethyl)-2-chloropyridine [21] (85 mg, 0.41 mmol) as described for compound **6**. Pale solid, 52 mg, yield 43%. ^1^H NMR (600 MHz, CDCl_3_) δ 8.36 (s, 1H), 8.32 (dd, *J* = 5.1, 0.7 Hz, 1H), 7.33–7.30 (m, 2H), 7.16 (d, *J* = 0.8 Hz, 1H), 7.06 (dd, *J* = 5.1, 0.7 Hz, 1H), 7.04–7.01 (m, 3H), 6.24 (s, 2H), 5.53 (s, 2H), 5.41 (s, 2H). HRMS (ESI^+^) m/z calcd for C_18_H_16_ClN_6_O [M + H]^+^ 367.1069, found 367.1078.

#### 4.2.41. 1-((6-Chloropyridin-3-yl)methyl)-3-(phenoxymethyl)-1H-pyrazolo[3,4-d]pyrimidin-4-amine (**21**)

Compound **21** was prepared from **47a** (80 mg, 0.33 mmol) and 5-(bromomethyl)-2-chloropyridine [21] (85 mg, 0.41 mmol) as described for compound **6**. White solid, 47 mg, yield 38%. ^1^H NMR (600 MHz, CDCl_3_) δ 8.45 (d, *J* = 2.5 Hz, 1H), 8.36 (s, 1H), 7.61 (dd, *J* = 8.3, 2.5 Hz, 1H), 7.31–7.29 (m, 2H), 7.26 (d, *J* = 8.3 Hz, 1H), 7.04–7.01 (m, 3H), 6.20 (s, 2H), 5.52 (s, 2H), 5.37 (s, 2H). HRMS (ESI^+^) m/z calcd for C_18_H_16_ClN_6_O [M + H]^+^ 367.1069, found 367.1070.

#### 4.2.42. 1-((2-Aminopyridin-4-yl)methyl)-3-(phenoxymethyl)-1H-pyrazolo[3,4-d]pyrimidin-4-amine (**22**)

TFA (2 mL) was added dropwise to a solution of **24** (29 mg, 0.065 mmol) in anhydrous CH_2_Cl_2_ (4 mL) at room temperature. The resulting mixture was stirred at room temperature for 1 h and then concentrated. The residue was treated with strong ammonia (1 mL) and then purified by flash column chromatography (0–15% MeOH/CH_2_Cl_2_) to give compound **22** as a white solid (22 mg, 98%). ^1^H NMR (600 MHz, DMSO-*d_6_*) δ 8.25 (s, 1H), 7.87 (d, *J* = 6.4 Hz, 1H), 7.68 (s, 2H), 7.33–7.30 (m, 2H), 7.07–7.05 (m, 2H), 6.98 (t, *J* = 7.2 Hz, 1H), 6.57 (d, *J* = 6.4 Hz, 1H), 6.45 (s, 1H), 5.53 (s, 2H), 5.44 (s, 2H). HRMS (ESI^+^) m/z calcd for C_18_H_18_N_7_O [M + H]^+^ 348.1567, found 348.1563.

#### 4.2.43. 1-((6-Aminopyridin-3-yl)methyl)-3-(phenoxymethyl)-1H-pyrazolo[3,4-d]pyrimidin-4-amine (**23**)

Compound **23** was prepared from **25** (53 mg, 0.12 mmol) as described for compound **22**. White solid, 30 mg, yield 73%. ^1^H NMR (600 MHz, DMSO-*d_6_*) δ 8.23 (s, 1H), 7.91 (s, 1H), 7.29–7.27 (m, 4H), 7.04–7.03 (m, 2H), 6.95 (t, *J* = 7.4 Hz, 1H), 6.36 (d, *J* = 8.5 Hz, 1H), 5.94 (s, 1H), 5.40 (s, 2H), 5.28 (s, 2H). HRMS (ESI^+^) m/z calcd for C_18_H_18_N_7_O [M + H]^+^ 348.1567, found 348.1572.

#### 4.2.44. Tert-Butyl (4-((4-Amino-3-(phenoxymethyl)-1H-pyrazolo[3,4-d]pyrimidin-1-yl)methyl)pyridin-2-yl)carbamate (**24**)

Compound **24** was prepared from **47a** (100 mg, 0.41 mmol) and *tert*-butyl (4-(bromomethyl)pyridin-2-yl)carbamate [22] (143 mg, 0.50 mmol) as described for compound **6** except that DMF was used a reaction solvent. White solid, 42 mg, yield 23%. ^1^H NMR (600 MHz, CDCl_3_) δ 8.36 (s, 1H), 8.12 (d, *J* = 6.5 Hz, 1H), 8.00 (s, 1H), 7.93 (s,1H), 7.32–7.27 (m, 2H), 7.04–7.02 (m, 3H), 6.59 (s, 1H), 6.30 (s, 2H), 5.54 (s, 2H), 5.41 (s, 2H), 1.52 (s, 9H). HRMS (ESI^+^) m/z calcd for C_23_H_26_N_7_O_3_ [M + H]^+^ 448.2092, found 448.2097.

#### 4.2.45. Tert-Butyl (5-((4-Amino-3-(phenoxymethyl)-1H-pyrazolo[3,4-d]pyrimidin-1-yl)methyl)pyridin-2-yl)carbamate (**25**)

Compound **25** was prepared from **47a** (100 mg, 0.41 mmol) and *tert*-butyl (5-(bromomethyl)pyridin-2-yl)carbamate [22] (143 mg, 0.50 mmol) as described for compound **6**. White solid, 72 mg, yield 39%. ^1^H NMR (600 MHz, CDCl_3_) δ 8.37 (s, 1H), 8.30 (d, *J* = 2.4 Hz, 1H), 7.97 (s, 1H), 7.89 (d, *J* = 8.7 Hz, 1H), 7.67 (dd, *J* = 8.7, 2.4 Hz, 1H), 7.31–7.28 (m, 2H), 7.02–7.00 (m, 3H), 6.27 (s, 2H), 5.47 (s, 2H), 5.37 (s, 2H), 1.51 (s, 9H). HRMS (ESI^+^) m/z calcd for C_23_H_26_N_7_O_3_ [M + H]^+^ 448.2092, found 448.2096.

#### 4.2.46. 1-(4-Nitrobenzyl)-3-((o-tolyloxy)methyl)-1H-pyrazolo[3,4-d]pyrimidin-4-amine (**26**)

Compound **26** was prepared from **47b** (45 mg, 0.176 mmol) and 4-nitrobenzyl bromide (46 mg, 0.21 mmol) as described for compound **6**. Pale solid, 47 mg, yield 68%. ^1^H NMR (600 MHz, CDCl_3_) δ 8.37 (s, 1H), 8.17 (d, *J* = 8.7 Hz, 2H), 7.44 (d, *J* = 8.7 Hz, 2H), 7.18 (dd, *J* = 7.5, 1.7 Hz, 1H), 7.14 (td, *J* = 7.5, 1.7 Hz, 1H), 6.99 (d, *J* = 8.1 Hz, 1H), 6.94 (td, *J* = 7.5, 0.9 Hz, 1H), 6.22 (s, 2H), 5.65 (s, 2H), 5.39 (s, 2H), 2.28 (s, 3H). HRMS (ESI^+^) m/z calcd for C_20_H_19_N_6_O_3_ [M + H]^+^ 391.1513, found 391.1519.

#### 4.2.47. 1-(4-Nitrobenzyl)-3-((m-tolyloxy)methyl)-1H-pyrazolo[3,4-d]pyrimidin-4-amine (**27**)

Compound **27** was prepared from **47c** (45 mg, 0.176 mmol) and 4-nitrobenzyl bromide (46 mg, 0.21 mmol) as described for compound **6**. White solid, 36 mg, yield 52%. ^1^H NMR (600 MHz, CDCl_3_) δ 8.36 (s, 1H), 8.16 (d, *J* = 8.7 Hz, 2H), 7.42 (d, *J* = 8.7 Hz, 2H), 7.17 (td, *J* = 7.6, 0.9 Hz, 1H), 6.85–6.81 (m, 3H), 6.27 (s, 2H), 5.64 (s, 2H), 5.38 (s, 2H), 2.31 (s, 3H). HRMS (ESI^+^) m/z calcd for C_20_H_19_N_6_O_3_ [M + H]^+^ 391.1513, found 391.1519.

#### 4.2.48. 1-(4-Nitrobenzyl)-3-((p-tolyloxy)methyl)-1H-pyrazolo[3,4-d]pyrimidin-4-amine (**28**)

Compound **28** was prepared from **47d** (45 mg, 0.176 mmol) and 4-nitrobenzyl bromide (46 mg, 0.21 mmol) as described for compound **6**. White solid, 43 mg, yield 62%. ^1^H NMR (600 MHz, CDCl_3_) δ 8.36 (s, 1H), 8.16 (d, *J* = 8.7 Hz, 2H), 7.41 (d, *J* = 8.7 Hz, 2H), 7.09 (d, *J* = 8.6 Hz, 2H), 6.91 (d, *J* = 8.6 Hz, 2H), 6.28 (s, 2H), 5.63 (s, 2H), 5.26 (s, 2H), 2.29 (s, 3H). HRMS (ESI^+^) m/z calcd for C_20_H_19_N_6_O_3_ [M + H]^+^ 391.1513, found 391.1522.

#### 4.2.49. 3-((2-Chlorophenoxy)methyl)-1-(4-nitrobenzyl)-1H-pyrazolo[3,4-d]pyrimidin-4-amine (**29**)

Compound **29** was prepared from **47e** (84 mg, 0.30 mmol) and 4-nitrobenzyl bromide (100 mg, 0.46 mmol) as described for compound **6**. Pale solid, 103 mg, yield 84%. ^1^H NMR (400 MHz, CDCl_3_) δ 8.37 (s, 1H), 8.17 (d, *J* = 8.6 Hz, 2H), 7.43 (d, *J* = 8.6 Hz, 2H), 7.39 (dd, *J* = 8.0, 1.6 Hz, 1H), 7.18 (td, *J* = 8.0, 1.6 Hz, 1H), 7.08 (dd, *J* = 8.0, 1.6 Hz, 1H), 6.97 (td, *J* = 7.7, 1.5 Hz, 1H), 6.24 (s, 2H), 5.64 (s, 2H), 5.48 (s, 2H). ^13^C NMR (100 MHz, CDCl_3_) δ 158.2, 157.1, 155.5, 152.7, 147.8, 143.7, 141.4, 130.8, 128.8, 128.2, 124.1, 123.1, 122.7, 114.1, 99.9, 65.7, 49.9. HRMS (ESI^−^) m/z calcd for C_19_H_14_ClN_6_O_3_ [M-H]^−^ 409.0821, found 409.0837.

#### 4.2.50. 3-((3-Chlorophenoxy)methyl)-1-(4-nitrobenzyl)-1H-pyrazolo[3,4-d]pyrimidin-4-amine (**30**)

Compound **30** was prepared from **47f** (45 mg, 0.16 mmol) and 4-nitrobenzyl bromide (53 mg, 0.24 mmol) as described for compound **6**. Pale solid, 41 mg, yield 63%. ^1^H NMR (400 MHz, CDCl_3_) δ 8.37 (s, 1H), 8.17 (d, *J* = 8.7 Hz, 2H), 7.43 (d, *J* = 8.7 Hz, 2H), 7.21 (t, *J* = 8.1 Hz, 1H), 7.25–6.97 (m, 2H), 6.90 (dd, *J* = 8.3, 0.9 Hz, 1H), 6.14 (s, 2H), 5.64 (s, 2H), 5.40 (s, 2H). ^13^C NMR (100 MHz, CDCl_3_) δ 158.0, 157.9, 157.0, 155.4, 147.8, 143.6, 141.5, 135.5, 130.8, 128.7, 124.2, 122.8, 115.6, 113.5, 99.8, 65.3, 49.9. HRMS (ESI^−^) m/z calcd for C_19_H_14_ClN_6_O_3_ [M-H]^−^ 409.0821, found 409.0839.

#### 4.2.51. 3-((4-Chlorophenoxy)methyl)-1-(4-nitrobenzyl)-1H-pyrazolo[3,4-d]pyrimidin-4-amine (**31**)

Compound **31** was prepared from **47g** (47 mg, 0.17 mmol) and 4-nitrobenzyl bromide (60 mg, 0.28 mmol) as described for compound **6**. Pale solid, 39 mg, yield 57%. ^1^H NMR (400 MHz, DMSO-*d_6_*) δ 8.23 (s, 1H), 8.17 (d, *J* = 8.8 Hz, 2H), 7.41 (d, *J* = 8.8 Hz, 2H), 7.33 (d, *J* = 9.0 Hz, 2H), 7.06 (d, *J* = 9.0 Hz, 2H), 5.65 (s, 2H), 5.43 (s, 2H). ^13^C NMR (100 MHz, DMSO-*d_6_*) δ 157.9, 156.5, 156.4, 154.7, 146.9, 144.6, 140.8, 129.2, 128.5, 125.0, 123.7, 116.9, 98.5, 63.8, 49.0. HRMS (ESI^−^) m/z calcd for C_19_H_14_ClN_6_O_3_ [M-H]^−^ 409.0821, found 409.0831.

### 4.3. Antiviral Assays

The ZIKV reporter assay and titer-reduction assay were performed as previously reported [17]. Briefly, Huh7 cells (1.5 × 10^4^) were seeded in a 96-well plate and inoculated with a luciferase reporter ZIKV (MOI=0.2) [17]. After two hours, the cells were treated with medium containing the compound (10 µM). After 3 days post-infection, the cells were lysed, the Nanoluc signal was detected using the Nano-Glo^®^ luciferase assay (Promega), then measured using a Neo 2 plate reader (BioTek, Winooski, VT, USA). For titer-reduction assays, 1.5 × 10^5^ Huh7 cell were seeded in a 24-well plate. Next day, the cells were inoculated with ZIKV for two hours. The inoculum was replaced with compound-containing medium (8.5 µM) and incubated for 3 days. Supernatants were collected and viral titers were estimated by plaque assay.

### 4.4. Cell Viability (CC_50_) Assay

The cell viability assay was performed in Huh7 cells as previously reported [17]. Briefly, non-infected Huh7 cells were treated with the testing compounds for 3 days. Viability was measured by absorbance at 490 mM using the MTS-based tetrazolium reduction CellTiter 96 Aqueous Non-Radioactive cell proliferation assay (Promega).

## Figures and Tables

**Table 1:** SAR study on the scaffold replacement.

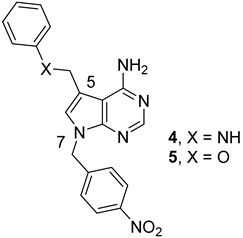							
**Compd**	**X**	**ZIKV Reporter Assay**			**Titer Reduction Assay**		**CC_50_ (µM)**
		**Inhibition (%) at 10 µM**	**Viability (%) at 10 µM**	**EC_50_ (µM)**	**% at 8.5 µM**	**EC_50_** **(µM)**	
**4**	NH	91	40				
**5**	O	62	82		94	4.3	58
DMSO		0	100		0		
NITD008 (1 µM) ^a^		100	100	0.14	100 (ND ^b^)		>45
NSC 12155 ^a^		68	70	2.18	72	7.34	23.0

^a^ Reported previously [17]; ^b^ ND, no plaque detected.

**Table 2:** SAR study on ring A of 1*H*-pyrazolo[3,4-d]pyrimidin-4-amine analogs.

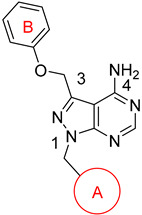							
**Compd**	**A**	**ZIKV Reporter Assay**			**Titer Reduction Assay**		**CC_50_ (µM)**
		**Inhibition (%) at 10 µM**	**Viability (%) at 10 µM**	**EC_50_ (µM)**	**% at 8.5 µM**	**EC_50_** **(µM)**	
**6**	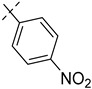	65	82		65	5.1	39
**7**	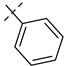	0	99				
**8**	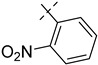	9	103				
**9**	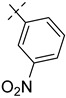	90	37				
**10**	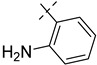	0	99				
**11**	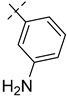	0	89				
**12**	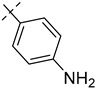	0	95				
**13**	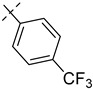	68	86		73	6.5	>100
**14**	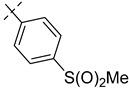	58	93		52		
**15**	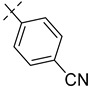	37	99				
**16**	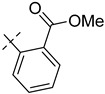	0	95				
**17**	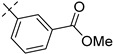	88	38				
**18**	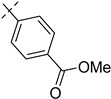	6	95				
**19**	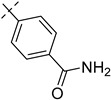	63	77		22		
**20**	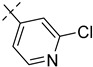	88	39				
**21**	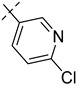	82	64	3.5	74	5.2	25
**22**	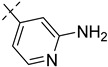	67	36				
**23**	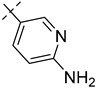	49	82		20		
**24**	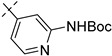	62	36				
**25**	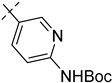	68	40				

**Table 3:** SAR study on ring B of 1*H*-pyrazolo[3,4-d]pyrimidin-4-amine analogs.

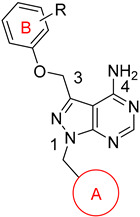								
**Compd**	**R**	**A**	**ZIKV Reporter Assay**			**Titer Reduction Assay**		**CC_50_ (µM)**
			**Inhibition (%) at 10 µM**	**Viability (%) at 10 µM**	**EC_50_ (µM)**	**% at 8.5 µM**	**EC_50_** **(µM)**	
**26**	*o*-Me	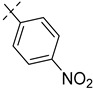	60	77				
**27**	*m*-Me	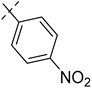	71	82		53		
**28**	*p*-Me	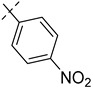	65	85		34		
**29**	*o*-Cl	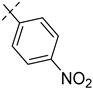	34	94				
**30**	*m*-Cl	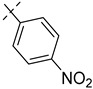	52	93		11		
**31**	*p*-Cl	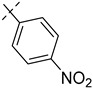	60	94		40		

## Data Availability

The data presented in this study and associated additional data are available upon request.

## Sample Availability

The reported compounds will be available to researchers under a Material Transfer Agreement.

## Abbreviations

DENV, dengue virus; DMF, dimethylformamide; JEV, Japanese encephalitis virus; MOI, multiplicity of infection; MTase, methyltransferase; RdRp, RNA-dependent RNA polymerase; SAR, structure-activity relationship; WHO, World Health Organization; WNV, West Nile virus; YFV, yellow fever virus; ZIKV, Zika virus.
